# Trichiniasis

**Published:** 1866-08

**Authors:** 


*Trichiniasis.*—We make the following extracts from an article in the St. Louis *Medical Recorder*, by Dr. J. H. Wilson, of Iowa, confirmatory of former reports on this subject, and intimating its probable identity with *hog cholera:*

Henry B. died June 3; Albert L., the 8th; Mr. B., the 15th; and Mrs. B., the 17th. *Post mortem* was held over Mr. Bemis, but practically no new facts were elicited. Trichinæ were discovered in as great abundance as in the muscles of the boy previously examined, some again having motion. Whitfield was able to ride out in the country on June 4. After their cases in town had been diagnosed trichiniasis, Dr. Ristine, one of the consulting physicians in these cases, came to the conclusion that some six persons, patients of his, residing in Maine township, this county, twelve miles north-east of Marion, who were affected precisely similar to these cases, were laboring under che same disease. The persons affected, three girls and three boys, aged respectively 16, 14, 14, 11, 9, 8, children of M. C. Jordan, W. Jordan, B. F. Jordan and Widow Daggett, on their way from Sabbath-school, April 22, stopped at a house and took a lunch of sandwiches, in which raw, smoked ham was used. Two days afterward, all but one of them were attacked with diarrhœa, developing into all the symptoms of trichiniasis. Fortunately, the parents of the children affected promptly administered to each of them an active dose of cathartic pills upon their showing the first symptoms of sickness. The eldest girl exhibited the symptoms in a reversed order—lameness of the muscles on the second, and diarrhœa on the third day. She was more severely affected than the others; had inflammation of the lungs, and, when seen by Dr. Ristine on the 19th, was scarcely able to walk, and had also a cough and hoarseness of voice. No cathartic was given her at first as to others. As anasarca was the most prominent symptom with the cases Dr. R. had to do with, he prescribed potass. bitart and jalap, combatting the symptoms as they arose on general principles. Portions of the same ham eaten by the family were subjected to microscopical examination, and were found to be swarming with myriads of the parasites. The same meat was eaten by the family to which it belonged, when well cooked, with no unhappy results. The hog was selected for its healthy appearance from a herd which was affected with hog cholera last fall, but, recovering, was fattened and killed for family use in January last. This, you see, forms another link in the chain of testimony, that trichiniasis and hog cholera are one and the same disease. Therefore, we think it behooves the medical profession to instruct the public of the connected nature of these two destructive maladies, in order that another such endemic as has occurred here may, as far as possible, be prevented.

